# Recycling of Waste Solution after Hydrothermal Conversion of Fly Ash on a Semi-Technical Scale for Zeolite Synthesis

**DOI:** 10.3390/ma14061413

**Published:** 2021-03-15

**Authors:** Rafał Panek, Jarosław Madej, Lidia Bandura, Grzegorz Słowik

**Affiliations:** 1 Department of Geotechnical Engineering, Civil Engineering and Architecture Faculty, Lublin University of Technology, Nadbystrzycka 40, 20-618 Lublin, Poland; j.madej@pollub.pl (J.M.); l.bandura@pollub.pl (L.B.); 2 Department of Chemical Technology, Institute of Chemical Sciences, Faculty of Chemistry, Maria Curie-Sklodowska University in Lublin, Maria Curie-Sklodowska Sq. 3, 20-031 Lublin, Poland; grzegorz.slowik@poczta.umcs.lublin.pl

**Keywords:** waste solution, zeolites, fly ash

## Abstract

Nowadays, using fly ash for zeolites production has become a well-known strategy aimed on sustainable development. During zeolite synthesis in a hydrothermal conversion large amount of post-reaction solution is generated. In this work, the solution was used as a substrate for Na-A and Na-X zeolites synthesis at laboratory and technical scale. Obtained materials were characterized using particle size analysis, X-ray diffraction (XRD), X-ray fluorescence spectroscopy (XRF), transmission electron microscopy (TEM), Fourier transformed infrared spectroscopy (FTIR), and nitrogen adsorption/desorption isotherm. Produced zeolites revealed high purity (>98%) and monomineral zeolitic phase composition. The SiO_2_ content was in the range 39–42% and 40–38%, whereas Al_2_O_3_ content was 23–22% and 25–26% for Na-X and Na-A, respectively. TEM and BET analyses revealed Na-X zeolite pores were almost identical to commercial 13X with S_BET_ in the range 671–734 m^2^/g. FTIR indicated slight differences between materials obtained at laboratory and technical scale in Si-O-(Si/Al) bridges of the zeolitic skeleton. The results showed good replicability of the laboratory process in the larger scale. The proposed method allows for waste solution reusability with a view to highly pure zeolites production in line with circular economy assumptions.

## 1. Introduction

Fly ash is one of the most common pollution in the world. It comes from combustion of coal in power plants and thermal power plants. Almost 45% of carbon emissions (energy related) is caused by coal combustion processes [1]. Currently, fly ash is most often deposited in landfills which leads to deterioration of the environment and human health [2]. The most common way of its disposal is using them in civil engineering (as an additive in cement industry) [3]. However, this application imposes certain limitations related to the chemical composition of the ash. Nowadays, new technologies introduced in power plants and thermal power plants have a negative impact on the chemical composition of fly ash, e.g., by increasing the content of unburned coal that excludes their use in the construction industry [2,4]. Fly ashes are comoposed of aluminosilicate glaze (65–78%) and a crystalline part in the form of mullite, quartz, and hematite, magnetite, or calcite [5,6]. Such composition (silica and alumina-rich) enables their use in the synthesis of porous aluminosilicate materials [7,8], in particular zeolites.

Zeolites are aluminosilicate minerals composed of [AlO_4_] and [SiO_4_] tetrahedrons (joint through corners) that build a 3D lattice of regular channels and chambers. Such framework provides zeolites with sorption, catalysis, ion-exchange or molecular sieving properties. Due to their practical features, zeolites are widely applied in environmental engineering [9], civil engineering [10,11], catalysis [12,13,14], and medicine [15,16,17].

Over 100 natural zeolites can be distinguished, among which only several types, like clinoptilolite or phillipsite, are accessible for mining and further use. On the contrary, over 150 types of synthetic zeolites have been obtained so far, using various substrates, for example chemicals [18], clay minerals [19,20,21], silicate minerals [22], rice husk [23,24], or wastes like fly ash (carbon combustion byproduct) [9,25,26,27]. The use of fly ash to produce zeolites is attracting many enthusiasts because that is one way to minimize waste and is beneficial to the environment. Therefore, obtaining zeolites from fly ash is quite widely reported in the literature.

Several methods can be applied for the synthesis of zeolites from fly ash. One of the most common is a hydrothermal conversion. It is based on the reaction of fly ash with alkaline solution at elevated temperature (up to 200 °C) for a specified period of time (from several to 72 h or sometimes more). The first results of alkaline treatment of coal combustion ash were published by Mondragon and Querol [28,29]. This method has been transferred to a technical scale [30] which allows to obtain large amount of zeolites during one synthesis cycle. Apart from zeolite phases, an unreacted residuum from fly ash is usually obtained that contains aluminosilicate glaze, mullite, and quartz. Moreover, the final product may consist a mixture of two or more zeolite types instead of a zeolite in a mono-mineral form [31,32]. In addition, hydrothermal process generates a post-reaction solution as a waste which is not further used.

Another known method is fusion, in which fly ash is mixed with solid sodium hydroxide and sintered at 550–600 °C. Then, the homogenous sinter is subjected to hydrothermal crystallization at elevated temperature [33,34]. Due to the sintering at high temperatures, this method is relatively costly and energy consuming. Therefore, the fusion method in technical or industrial scale is not economically justified.

Hollman et al. [35] proposed a two-step synthesis method to obtain pure zeolite phases. The first step involves the hydrothermal reaction of fly ash with a sodium hydroxide solution to produce a filtrate rich in silicon and aluminum. The fly ash after the treatment is filtered and discarded. There are also known two-step syntheses in which fusion or sonication are used as a first step [25,34]. In the second step, the filtrate solution is modified to obtain an appropriate Si/Al ratio and subjected to further hydrothermal conversion to produce final pure zeolite. However, these trials have only been conducted on a laboratory scale [36]. The disadvantage of this method is generation of solid waste in the form of fly ash after the first-step treatment.

Different types of zeolite structures can be obtained synthetically, including those from gismondine, faujasite, linde A, or sodalite groups. Taking into account attractive properties and potential applications, NaX (faujasite, FAU) and NaA (Linde A, LTA) are very popular zeolite structures. The FAU structure is characterized by a double six-ring (D6R) skeleton. It is composed of sodalite units constituting the β-chambers, which are connected with each other by hexagonal prisms (double six-membered ring D6R). This arrangement of structural units creates a second type of chambers called supercells with a diameter of about 1.3 nm, which in turn are connected with each other by four 12-membered rings with a diameter of about 0.74 nm, that are the internal pores of the crystal structure, according to the International Zeolite Organization—International Zeolite Association. In turn, the LTA structure has a double four-ring (D4R) skeleton. It is formed by cubooctahedral sodalite units (β chamber) with a diameter of 0.66 nm, connected to each other by means of double four-membered D4R rings. In the spatial structure of the LTA structure, each cubooctahedron is connected with six others, creating a space called a large α chamber with a diameter of 1.14 nm, and six eight-membered rings with a diameter of 0.42 nm leading into it [37].

Thanks to the unique structure and the channel sizes zeolites Na-X and Na-A exhibit good adsorption and catalytic properties and have been studied in terms of CO_2_ adsorption [23,38,39], heavy metals removal [31,40,41,42], in biomass thermal processes towards hydrogen-rich gas [43], transesterification of mustard oil process [44], as well as dental fillers [45].

To date, no studies have been published on the use of waste solution after the synthesis of zeolites from fly ash to produce pure zeolite materials. Several reports concern the usage of a solution after fly ash extraction in a two-step method of pure zeolite synthesis. However, during this process, the fly ash waste is not intended to obtain the zeolite product but is discarded, and the solution with extracted aluminum and silicon is further used for zeolite synthesis in a laboratory scale.

The aim of this study was to synthesize zeolites with Na-X and Na-A structures from a post-reaction solution on the laboratory and technical scale. The solution is derived as a waste after the production of zeolites from fly ash in a hydrothermal conversion process on a technological demonstration line. According to current state-of-the-art, the usage of post-production waste solution for synthesis of ultrapure zeolites has not been studied yet. Hence, the originality of such an idea is unquestionable. The two zeolite products are obtained during one cycle of synthesis—first when waste solution is generated (zeolitic material) and second when waste solution is used as a raw substrate (ultrapure zeolite). Additionally, increasing the scale of the synthesis process opens the door to the industrial use of this type of material. Hence, our idea was to recycle the solution obtained directly after full-fledged synthesis of zeolites from fly ash. To the best of our knowledge, such an activity has been undertaken for the first time. An additional element of novelty of the presented research is the transfer of the synthesis process from the laboratory scale to the technical scale. The designed technical-scale synthesis parameters open the door to the industrial production of ultrapure zeolites as the eco-friendly and alternative way to the conventional methods based on chemical reagents usage.

## 2. Materials and Methods

### 2.1. Materials

Waste solution used for the production of ultrapure Na-X and Na-A zeolites was obtained after the hydrothermal synthesis of zeolites using a technological line described by Wdowin et al. [30]. Briefly, 20 kg of fly ash (Kozienice Power Plant, Kozienice, Poland) was mixed with 90 L of 3 M sodium hydroxide solution. The mixture was subjected to mechanical stirring and hydrothermal conversion lasted 48 h at a temperature of 80 °C in a stainless steel reaction tank. The solid product (in this case zeolitic material with fly ash residuum) was filtered and waste solution after the synthesis cycle was collected. Main chemical components of the solution were as follows: Na (19,433 mg/L), Si (13,711 mg/L), Al (36 mg/L). Aluminum foil (>99% Al, 0.25 mm, Pol-Aura, Zabrze, Poland) was used as Al source to achieve appropriate Si/Al ratio in the reaction system.

### 2.2. Synthesis

Two types of zeolites (Na-X and Na-A) were prepared from waste solution in two scales (laboratory—LS, and technical—TS) in a hydrothermal conversion. Hence, names of the samples are the following: Na-X-LS, Na-X-TS, Na-A-LS, and Na-A-TS. Specific synthesis parameters and the mass of the product obtained in one synthesis cycle are shown in Table 1.

In order to obtain zeolites on a laboratory scale, appropriate amount of NaOH aqueous solution was prepared in 250 mL polypropylene bottles. Then, the source of aluminum in the form of aluminum foil was added to the bottles and mixed until completely dissolved. In the last step, the waste solution was added to the mixture, and the bottles were transferred to a dryer in order to conduct a hydrothermal reaction at elevated temperature. Zeolites on a technical scale were obtained using a secondary-stage reactor, made of polypropylene with an operating volume of 100 L. The reactor is equipped with a temperature control and agitation system, therefore in this case the reaction mixture was not placed in the dryer but it was subjected to hydrothermal conversion by heating the reactor to a specific temperature. The synthesis was carried out using the amounts of reactants and parameters given in Table 1. Syntheses were conducted in an atmospheric pressure.

For both scales, the quantities of reagents were calculated considering silica content in waste solution. The amount of aluminum reactant was determined by the silicon content in the solution so that their ratio was at least 1.45 for Na-X and approximately 1.00 for Na-A. Taking into account economic aspects, tap water was used in the case of the technical scale.

Zeolites obtained both on laboratory and technical scale were rinsed with distillate water until the leakage reached pH 9, at which point it was filtered and dried at 105 °C for 24 h. Zeolite samples were collected and kept in a desiccator before further analyses.

### 2.3. Characterization Methods

Waste solution composition was determined using Inductively coupled plasma mass spectrometry (ICP-MS) on Agilent 8900 Triple Quadrupole apparathus (Agilent, Santa Clara, CA, USA).

Particle size analysis of obtained zeolites was performed by Mastersizer 3000 apparatus HYDRO EV add-on (Malvern Panalytical, Malvern, UK) using laser diffraction technique with a measurement range of 0.01 μm to 2 mm. Mie theory was applied during measurements.

Analysis of chemical composition of zeolites and fly ashes was conducted using semi-quantitative energy dispersive X-ray fluorescence (ED-XRF) method on Epsilon 3× (Panalytical, Eindhoven, The Netherlands) apparatus. The samples were used in their original powdered form in the amount of 3 g per sample.

The mineral/phase composition of zeolites was determined using the X-ray diffraction powder method (XRD) using a X’pert MPD X-ray diffractometer (Panalytical, Eindhoven, Netherlands) with a goniometer PW 3020 and a Cu lamp and a graphite monochromator. Diffraction patterns were recorded by step scanning from 5 to 65, with a step size of 0.02° and HighScore Pro software was used to process diffraction data. The identification of mineral phases was based on the PCPDFWIN ver. 1.30 formalized by JCPDS-ICDD. Before measurement, the samples were milled and sieved using 63 µm sieve.

For microscopic analyses the samples were grinded in an agate mortar to a fine powder. The resulting powder were poured with 99.8% ethanol (POCH) to form a slurry. The samples were inserted into the ultrasonic homogenizer for 10 s. Then, the slurry containing the samples was pipetted and supported on a 200 mesh copper grid covered with lacey formvar and stabilized with carbon (Ted Pella Company, Redding, CA, USA) and left on the filter paper until the ethanol evaporated. Subsequently, the samples deposited on the grid were inserted to holder individually and moved to the electron microscope. The electron microscope, Titan G2 60–300 kV FEI Company, Hillsboro, OR, USA, equipped with: field emission gun (FEG), monochromator, three condenser lenses system, the objective lens system, image correction (Cs-corrector), HAADF detector and EDS spectrometer (Energy Dispersive X-Ray Spectroscopy) EDAX Company with Si(Li) detector was used to display the prepared samples. Microscopic studies of the samples were carried out at an accelerating voltage of the electron beam equal to 300 kV. The mapping was carried out in the STEM mode by collecting point by point EDS spectrum of each of corresponding pixels in the map. The collected maps were presented in the form of a matrix of pixels with the color mapped significant element and the intensity corresponding to the percentage of the element.

Fourier transformed photoacoustic infrared spectra (FT-IR/PAS) of the examined samples were measured in the range of 4000–400 cm^−1^ at a room temperature, resolution of 4 cm^−1^, mirror velocity of 2.5 kHz, and maximum source aperture by FT-IR Excalibur 3000 spectrophotometer (Bio-Rad, Hercules, CA, USA) using an MTEC Model 300 photoacoustic cell.

The textural parameters of the studied zeolites were determined by means of the low-temperature nitrogen adsorption/desorption isotherm. The measurements were performed in a ASAP 2020 instrument (Micromeritics, Norcross, GA, USA). The samples were previously outgassed at 250 °C for 24 h under high vacuum. The specific surface areas, S_BET_, were calculated using the standard Brunauer–Emmett–Teller (BET) equation for nitrogen adsorption data in the range of relative pressure p/p_0_ from 0.05 to 0.30. The micropore area and volume (S_mic._ and V_mic._) were estimated by t-plot method. The mesopore area and volume (S_mes._ and V_mes._) were calculated from BJH theory.

## 3. Results and Discussion

### 3.1. Zeolites Particle Size Distrubution

Percentage distribution of individual grain fractions is presented in Table 2. 

**Figure 1:**
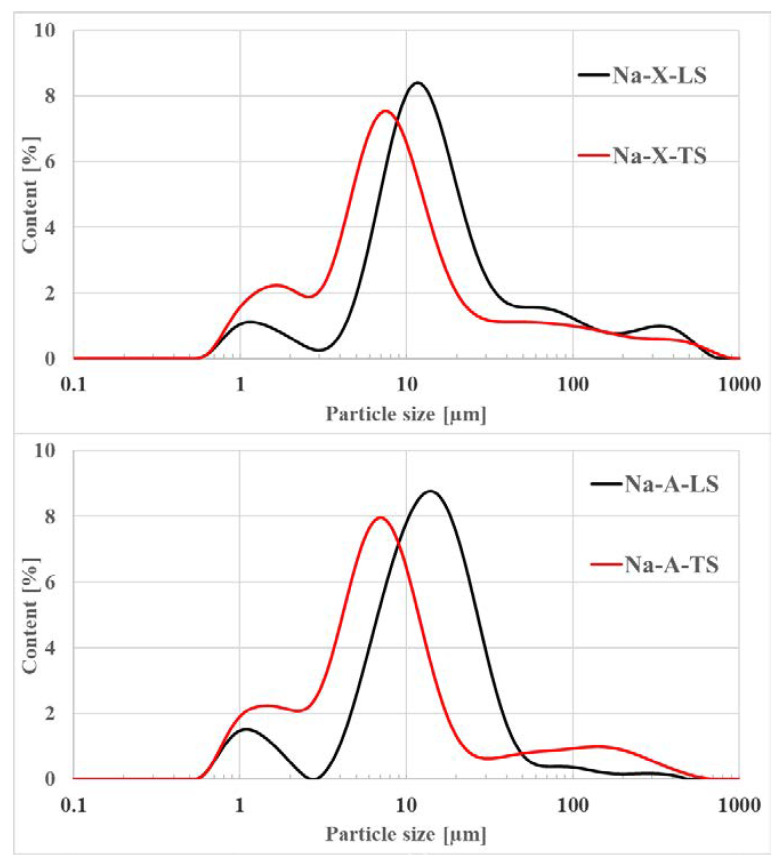
Particle size distribution curves for studied zeolites.

The most dominant fraction in the obtained zeolites was 2–20 μm (60.97–70.87% of the volume). It can be noticed that technical scale led to an increase in the proportion of this fraction by several percent compared to materials obtained on laboratory scale. Grains in the range of 20–50 μm constituted 16% of Na-X-LS and 21.86% of Na-A-LS. On the contrary, this fraction occurred in a minor amount in materials obtained on technical scale. Larger grains were a small percentage of the total volume of the materials. It is worth noting that the smallest fraction 0.01–2 μm occurs in all zeolites in the range of 6.26–8.22% for Na-X-LS and Na-A-LS respectively, and in the range of 11.86–13.22% for Na-X-TS and Na-A-TS respectively. That revealed Na-X zeolites were more fine powders that Na-A zeolites and the technical scale favored obtaining materials with smaller grain sizes in comparison with the materials derived on laboratory scale. Figure 1 shows particle size distribution curves for the obtained materials. Both Na-X and Na-A zeolites were characterized by bi- or trimodal particle distribution. For Na-X two clear maxima and one blurry were observed. First one was around 1 μm, second and the biggest was between 5–10 μm. The last one was the smallest and hard to see—at 500 μm. Generally, approximately 67–79% of the Na-X zeolites constituted the particles below 20 μm. Similar situation can be observed for Na-A particle size distribution curves where 74–84% of the samples were those diameter below 20 µm. Again, three maxima can be observed (two clear and one blurry). First and the biggest was noted for 1.5 μm, second for approximately 7–20 μm and the third one—200 μm. This results corresponded well to the values presented in Table 2. Technical scale led to smaller grain sizes of the produced materials. All zeolites are very fine powders. In contrary, Bandura et al. [46] revealed that Na-X obtained directly from fly ash exhibited uniform particle size distribution with a major contribution of fraction around 40 µm. In terms of particle size, zeolites from fly ashes possess larger particle sizes that can be explained by the fact that fly ash particles act as templates for zeolite nucleation, whereas pure zeolites are produced from solution in which small nuclei of crystallization in the form of silicates and alumina particles occur.

### 3.2. XRF Chemical Composition

The chemical composition of zeolites is presented in Table 3. It can be observed that in all materials the SiO_2_ and Al_2_O_3_ were the major components. Silica oxide was ranged from 39.3 to 41.7% for Na-X and 38.3 to 40.5% for Na-A zeolites while alumina oxide content did not exceed 25.9% and it was slightly higher for zeolites Na-A. The sodium oxide content was high (from 6.8 to 14.7%). It was due to the using sodium hydroxide as a main chemical reagent during the synthesis. Additionally, sodium element can be incorporated quite easy into zeolite structure. There was no sulfur or phosphorous detected. The concentrations of other oxides like K_2_O and Fe_2_O_3_ were quite low (1.2 and 1.3%, respectively). Additionally, MgO was determined only for zeolites produced on a technical scale. Similar situation can be noted for CaO where definitely higher content is observed for zeolites obtained in technical scale. This was due to the fact that tap water, instead of distillate one, was used for technical scale zeolites (for preparing NaOH solution). It should be noted that the loss of ignition (LOI) for all obtained zeolites was high (higher for zeolites Na-X than Na-A). This was due to the fact that zeolites have in their structure large amounts of adsorbed water, which they release at temperatures higher than 100 °C. It is worth mentioning that chemical composition of commercial Na-X and Na-A zeolites is similar to those obtained from waste solution (Table S1). There are some differences like CaO and MgO contents but only for TS zeolites which is a result of tap water usage during the synthesis. Taking into account chemical composition of zeolites from Faujasite and LTA types, different content of main components can be observed. However, an important parameter is the Si/Al molar ratio that is generally attributed to the zeolite type. Other components may be different depending on the substrates and synthesis methods used. In this study Si/Al molar ratios for Na-X was 1.51 and 1.48 for LS and TS, respectively. Si/Al molar ratio for Na-A-LS was 1.32, whereas for Na-A-TS it was 1.24. It can be noticed that the application of technical scale lowered slightly the Si/Al ratios of produced zeolites in relation to the laboratory scale. Nevertheless, the changes were very minor, in the range of 1–3%. Obtained values of Si/Al molar ratios are in good agreement with those published for zeolites of Na-X and Na-A types. In comparison, Bandura et al. reported fly ash-derived Na-X zeolite with 34.5% of SiO_2_ and 19.75% of Al_2_O_3_ (Si/Al ratio = 1.54) [47]. Another research showed that zeolite Na-A obtained from kaolinite had 32.38% of SiO_2_ and 28.05% of Al_2_O_3_ with Si/Al molar ratio 0.98 [48]. The obtained results of the chemical composition indicate the correct Si/Al molar ratio in the case of Na-X zeolite (both laboratory and technical scale). However, in the case of Na-A zeolite, the obtained Si/Al molar ratios were slightly higher than those found in the literature (ideally it should be around 1.00). This can be explained by the fact that waste solution is rich in silicon. 

### 3.3. XRD Mineralogy Characterization

**Figure 2:**
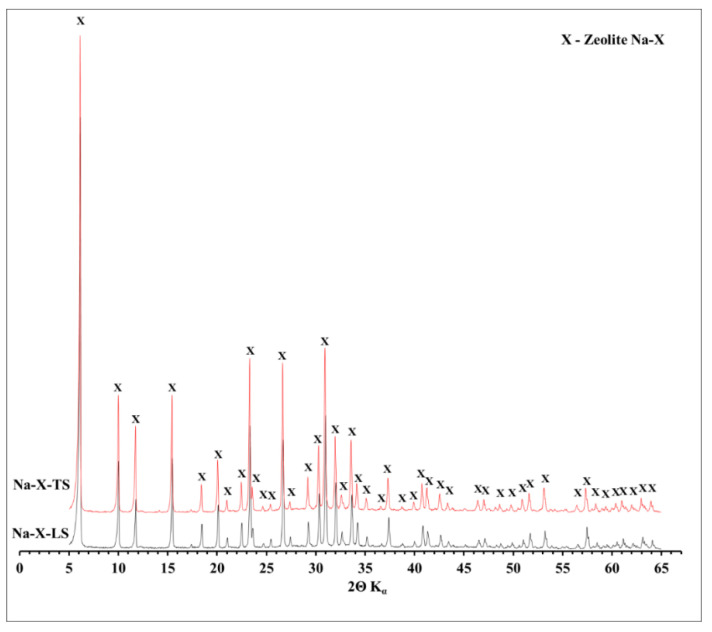
Mineral phase composition of Na-X-LS and Na-X-TS zeolites.

**Figure 3:**
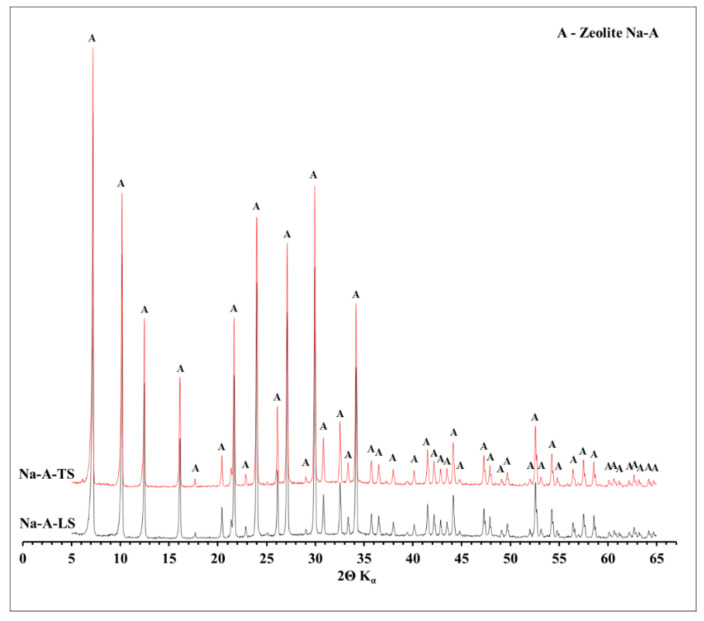
Mineral phase composition of Na-A-LS and Na-A-TS zeolites.

The phase composition determined by X-ray diffraction (XRD) of all tested zeolites is shown in Figure 2 and Figure 3. Characteristic reflexes were identified for all tested zeolites. For Na-X (both LS and TS) these were, among others: d_hkl_ = 14.45; 8.85; 3.34; 2.89 and 2.79 Å, while for Na-A: d_hkl_ = 12.23; 8.65; 3.70; 3.28; 2.98 Å. No other reflexes, apart from zeolite phases, were observed. The zeolites were characterized by monomineral zeolitic phases of high purity. The XRD results are in good agreement with the diffractograms of Na-X and Na-A commercial zeolites (Figures S1 and S2). On their diffractograms only specific, for Na-X and Na-A zeolites, d_hkl_ reflexes can be observed. Such comparison is a proof that waste solution can be successfully used for ultrapure zeolite synthesis. The shape, height, and reflection pattern on the diffractograms of the obtained zeolites and their commercial counterparts confirm the high degree of crystallinity of these materials. In addition, it can be observed that the diffractograms registered for two different scales were almost identical, which proves the repeatability of the synthesis regardless of the amount of raw materials used for the synthesis. It confirms that increasing the scale of synthesis was possible and successfully performed for both Na-X and Na-A zeolites. Moreover, the diffractograms did not register a background line elevation in the angular range of 15–35° 2θ, which is a very characteristic phenomenon for zeolites obtained from fly ash, attributed to the presence of an amorphous phase [49]. Importantly, on the diffraction patterns of fly ash-derived zeolites, apart from the higher background level, characteristic reflexes of fly ash phases such as mullite, quartz, calcite, or hematite were also observed [50]. This phenomenon occurs because the hydrothermal conversion of the fly ash does not proceed with 100% efficiency. During the hydrothermal process, amorphous silica and some of quartz and mullite dissolve to some extent and then the zeolite synthesis begins. The remaining mineral phases stay as unreacted residuum. The zeolites tested in this study are devoid of such phases, because, in a contrary to fly ash-derived zeolites, the waste solution is free from mineral solid phases.

### 3.4. TEM

**Figure 4:**
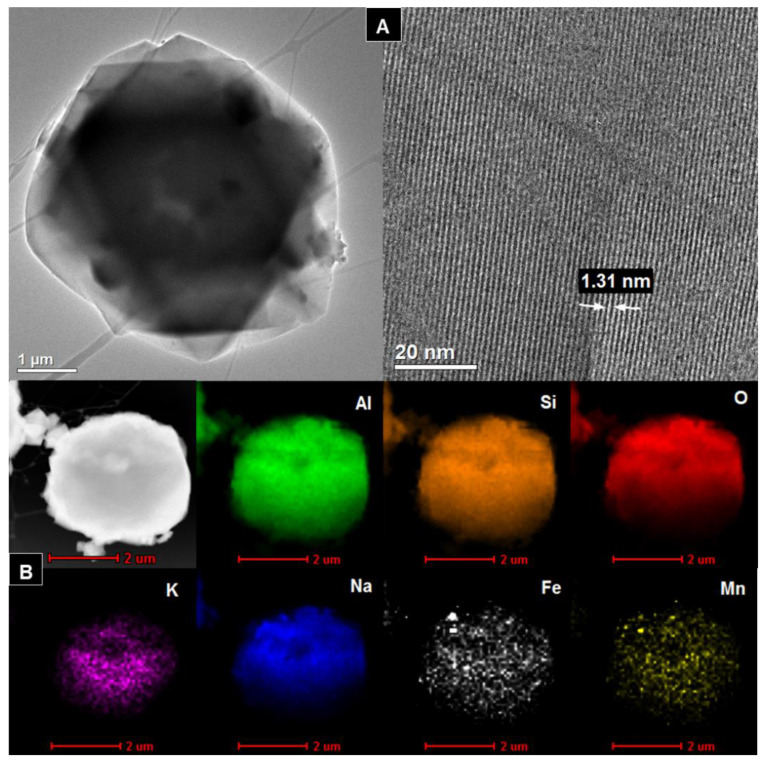
(**A**) HR/TEM images and (**B**) STEM-EDS analysis of Na-X-LS sample.

**Figure 5:**
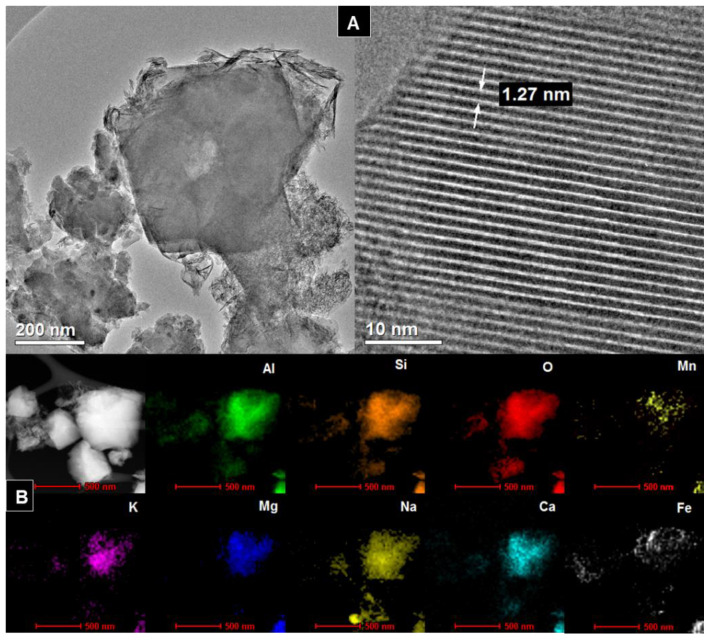
(**A**) HR/TEM images and (**B**) STEM-EDS analysis of Na-X-TS sample.

**Figure 6:**
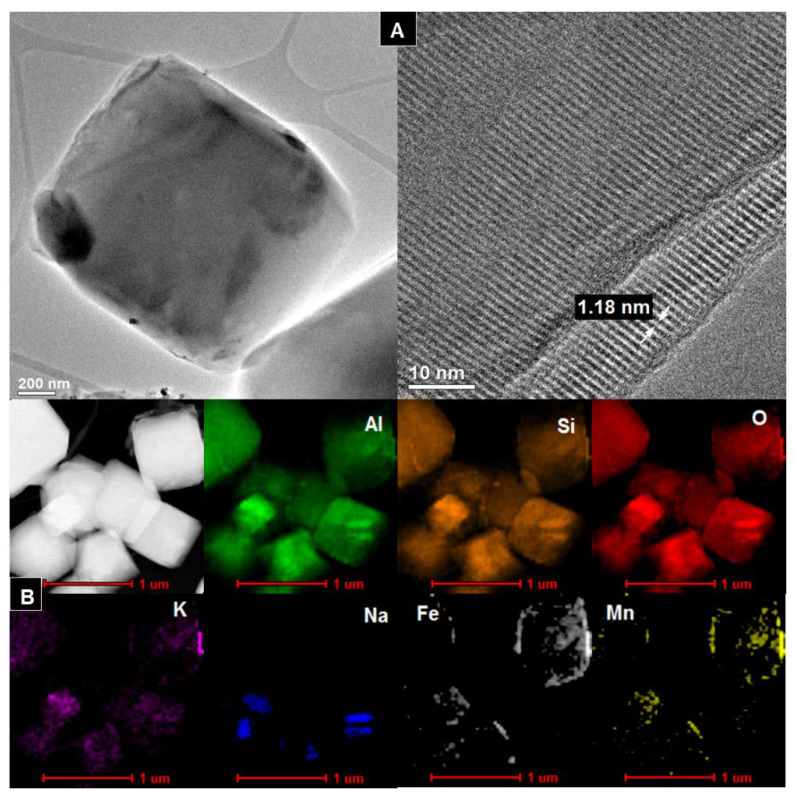
(**A**) HR/TEM images and (**B**) STEM-EDS analysis of Na-A-LS sample.

**Figure 7:**
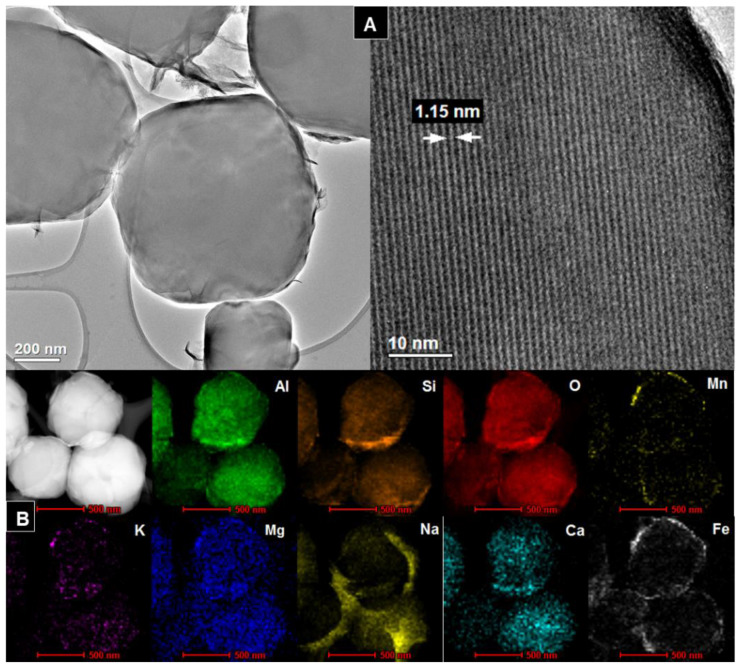
(**A**) HR/TEM images and (**B**) STEM-EDS analysis of Na-A-TS sample.

Figure 4A, Figure 5A, Figure 6A, Figure 7A show HR/TEM images of the Na-X-LS, Na-X-TS, Na-A-LS, and Na-A-TS samples. The structure of the analyzed materials was characterized by well ordered, hexagonal arrangement of cylindrical micropores with different diameters. The walls of the materials consisted mainly amorphous silica. Regular shape of the pores being cylindrical capillaries of the same size and regular arrangement can be observed in all analyzed samples. The particles had an irregular and various shape and size with visible regular pores characterized by a different diameter. The largest pore diameter, 1.31 nm, was recorded for the Na-X-LS sample. Na-X-TS showed pore diameter about 1.27 nm. Two pore diameters, 0.57 and 1.18 nm; and 0.84 and 1.15 nm, were observed for Na-A-LS and Na-A-TS, respectively. The STEM-EDS analysis (Figure 4B, Figure 5B, Figure 6B, Figure 7B) shows that the qualitative and quantitative composition of the elements in the individual samples was very similar with significant amount of silicon, aluminum and oxygen. These elements constituted the main particle structure of the samples. Significant amount of sodium was also noted, which came from the reagents used to synthesize these materials and it was incorporated into the structure of the samples. Small amounts of manganese, potassium, calcium, and iron were also detected. The qualitative composition of the particles of Na-X-TS sample (Figure 5B) included such elements as: Al, Si, Fe, K, Na, O, Mg, Mn, and Ca. The content of individual elements in the tested fragment of the sample was as follows: Al—16.65%, Si—26.15%, Fe—2.63%, K—0.56%, Na—3.54%, O—43.66%, Mg—2.27%, Mn—0.71%, Ca—3.78%. All of the elements were evenly dispersed in the sample.

The chemical composition of Na-A-TS was similar to that in Na-X-TS. The sample contained the following elements: Al—17.03%; Si—21.82%; O—50.84%; Mn—0.21%; K—0.33%; Mg—0.92%; Na—6.73%; Ca—1.04 %; Fe—1.03%. All elements were well dispersed in the samples. The significant amounts of iron, manganese and sodium can be observed on the surface of the grains of Na-A-TS. In turn, STEM-EDS analysis showed that the samples marked as Na-A-LS and Na-X-LS exhibited identical qualitative composition of elements. Both samples consisted the following elements composition: Al, Si, O, K, Na, Fe, and Mn, and all of them were well dispersed in the samples. Nevertheless, the content of elements in both samples was different. The content of individual elements in the analyzed fragment of Na-A-LS (Figure 6B) was: Al—20.74%; Si—21.55%; Fe—0.99%; K—0.63%; Na—8.67%; O—47.14%; Mn—0.26%. While the content of individual elements in the tested fragment of Na-X-LS (Figure 4B) was as follows: Al—24.36%; Si—26.69%; O—37.17%; K—0.93%; Na—10.55%; Fe—0.19%; Mn—0.07%.

### 3.5. FT-IR

**Figure 8:**
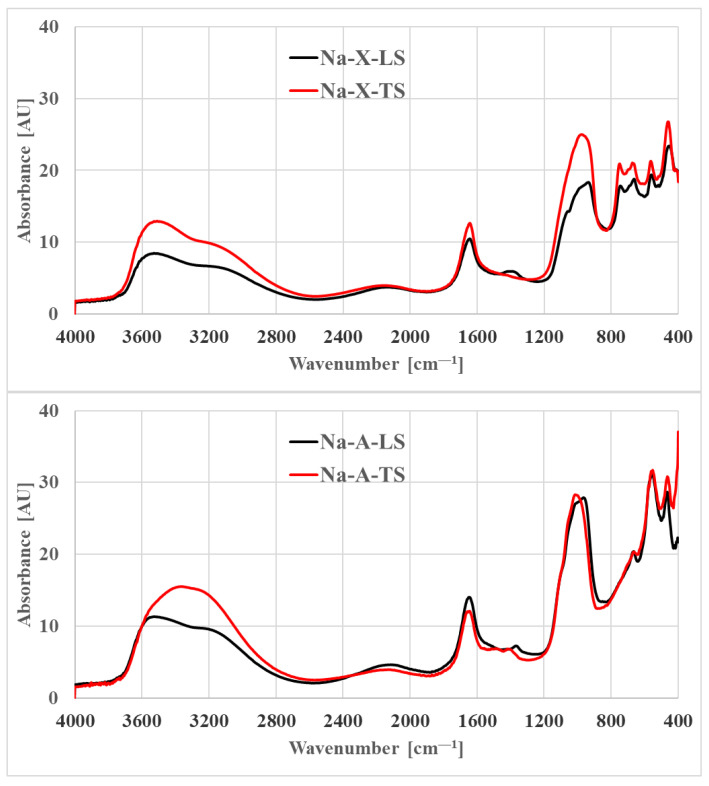
FT-IR spectra of investigated zeolites.

Figure 8 shows the FT-IR spectra of studied zeolites. The spectra of Na-A and Na-X samples obtained at both scales were characterized by bands typical for zeolite A (LTA) and faujasite (FAU) structures [51]. The bands with a maximum at about 3700–3500 cm^−1^ are associated with stretching vibrations of -OH groups on the materials surface. The band for Na-A-TS was smooth with the maximum at around 2400 cm^−1^, while the band for Na-A-LS was composed of two shoulders with the maxima at 3600 cm^−1^ and 3200 cm^−1^. For Na-X samples, analogical bands had a similar shape with two maxima, indicating the same nature of the surface -OH groups. In both cases (TS and LS), the intensity of the band was higher for materials synthesized at the technical scale, which may indicate a higher water content on their surface relative to materials obtained at the laboratory scale. Another characteristic band present at a wave number of about 1646 cm^−1^ is attributed to -OH groups bending vibrations of so called ‘zeolitic water’ and was present in all analyzed materials.

For Na-X-LS, a small band originating from carbonate vibrations was recorded at 1394 cm^−1^. The Na-X-TS did not show the presence of this band. On the other hand, it was present in both Na-A materials at 1377 cm^−1^. The bands in the range of about 1090–900 cm^−1^ are related to the asymmetric stretching vibrations of the Si-O(Al) and Si-O(Si) bridges. For the materials obtained in the laboratory scale, the maxima of these bands were slightly shifted towards lower wavenumbers and additional band around 920 cm^−1^ can be distinguished. The spectra for TS and LS materials indicate the possibility of differences in the Si/Al ratio in zeolites resulted from the process scale applied. The amount and a type of exchangeable cations can also be considered, which may affect the nature of the registered bands [52]. For Na-A, the band at 667 cm^−1^ is related to the symmetric stretching vibrations of the Si-O-Al bridges. The band at 556 cm^−1^ can be attributed to the symmetric Si-O-Si stretching and O-Si-O bending vibrations of the LTA 4-member ring [53]. For Na-X in the pseudolattice range, three distinct groups of bands can be distinguished. The first one in the range 740–690 cm^−1^, can be attributed to symmetric stretching vibrations of the four-member ring. These vibrations are accompanied by vibrations of the six-member ring, which are located at slightly lower wave numbers (670–560 cm^−1^). The third group of bands, with a maximum at 459 cm^−1^ is associated with O-Si(Al)-O bending vibrations of six-membered rings in D6R units or possibly with vibrations of higher-membered (12-membered) rings. For Na-X, the bands at 468 cm^−1^ are derived from O-Si-O bending vibrations.

### 3.6. ASAP 2020

Textural parameters of studied zeolites are presented in the Table 4. The zeolites from the FAU and LTA groups are microporous materials. It can be observed that in the case of Na-X zeolite, the specific surface area value (S_BET_) was at a similar level regardless of the production scale and ranged from 671 to 734 m^2^/g. The area of micropores in both cases was over 93%. Its commercial form has S_BET_ in a similar range—646 m^2^/g (Table S2). In this case the area of micropores is about 95% of all surface. This is another proof of similarity of Na-X structures regardless of its origin (waste or reagents). So far, we have obtained Na-X structures directly from fly ash with S_BET_ in the range of 157–236 m^2^/g [46,47], while Babajide et al. obtained S_BET_ of 320 m^2^/g for Na-X zeolite from South African waste coal fly ash [49]. Moreover, the S_BET_ value for zeolites obtained in the two-stage method (with calcination) achieved 257 m^2^/g [39]. The specific surfaces areas of Na-X zeolite obtained in this study are comparable, or even higher than those attributed to the highest class of zeolites obtained from chemical reagents or their commercial equivalents like 13X [54]. The low S_BET_ values for both the LS and TS Na-A zeolites is associated with the smaller pore size compared to the Na-X zeolites. The kinetic diameter of the nitrogen molecule (3.64 Å) is comparable to the effective channel diameter in the obtained Na-A zeolites (3–4 Å), so the situation in which these channels are blocked for the nitrogen molecule may occur. TEM studies showed a reduced channel size in Na-A zeolites (Figure 6 and Figure 7). On the other hand, an increased value for the mesopores (S_mes._) may result from agglomeration of zeolite crystals. The dosed gaseous nitrogen may enter the spaces between adjacent crystals and give the result of an apparent porosity increasing the specific surface area and the surface area of the mesopores [8,55]. Overall, the textural parameters demonstrate a high degree of similarity between the zeolites from waste and the high-grade zeolites obtained from chemical reagents (Table S2). Interestingly, obtained zeolites exhibited a slightly higher specific surface area than their commercial counterparts. This suggests that presented technology provides a product comparable in quality to superior zeolites prepared from chemical reagents.

## 4. Conclusions

In this paper, a sustainable and ecological route of the post-synthesis waste solution utilization in ultra-pure zeolite production has been presented for the first time. The solution after zeolite synthesis can be used as a valuable source of silica and alumina for the production of aluminosilicate solid materials with high quality, purity, and excellent surface properties. By optimizing the synthesis conditions the laboratory scale was successfully transferred to a technical one. Instrumental analysis confirmed that the textural, chemical, mineralogical, and morphological properties are very similar for materials obtained at both scales that suggests this process may be applied in an industrial practice. The waste solution can replace chemical reagents in the production of ultrapure zeolite materials according to cleaner production objectives. This method of producing pure zeolites from waste solution can significantly reduce production costs due to lower consumption of water, or aluminum and silicon sources. Thus, it can constitute a low-cost and environmentally friendly alternative to zeolite synthesis from chemical reagents. Obtained zeolites can find applications as the green and fully waste-derived adsorbents or catalysts in many branches of innovative, zero-waste economy.

## Figures and Tables

**Table 1:** Synthesis conditions of studied zeolites.

Zeolite	NaOH Conc. (M)	V_NaOH_ (dm^3^)	Al Source (g)	V_was.sol._ (dm^3^)	Time (h)	Temperature (°C)	Product Mass (g)
Na-X-LS	3	0.1	0.9	0.1	24	70	3
Na-X-TS	3	50	450	50	24	70	700
Na-A-LS	3	0.1	1.3	0.1	24	80	5
Na-A-TS	3	50	660	50	24	80	1180

**Table 2:** Percentage distribution of individual grain fractions (μm) of studied zeolites

Fraction (µm)	Na-X-LS	Na-X-TS	Na-A-LS	Na-A-TS
	(% Volume)			
0.01–2	6.26	11.86	8.22	13.23
2–20	60.97	67.31	66.09	70.87
20–50	16.00	7.68	21.86	4.51
50–100	6.68	4.84	1.94	3.89
100–250	5.33	4.80	1.32	5.51
250–500	4.08	2.60	0.58	1.87
500–1000	0.68	0.91	-	0.12
1000–2000	-	-	-	-

**Table 3:** Synthesis conditions of studied zeolites

Component	Na-X-LS	Na-X-TS	Na-A-LS	Na-A-TS
	(%)			
**Na_2_O**	12.42	6.78	14.69	10.76
**MgO**	nd	1.47	nd	0.62
**Al_2_O_3_**	23.15	22.42	25.43	25.86
**SiO_2_**	41.65	39.34	40.45	38.26
**K_2_O**	1.22	0.69	0.98	0.63
**CaO**	0.05	7.58	0.02	4.31
**TiO_2_**	0.02	0.04	0.02	0.03
**Fe_2_O_3_**	0.52	0.93	0.58	1.27
**LOI**	20.83	20.75	17.66	17.86

**Table 4:** Textural parameters of obtained zeolites.

Zeolite	S_BET_ (m^2^/g)	S_mic._ (m^2^/g)	S_mes._ (m^2^/g)	V_mic._ (cm^3^/g)	V_mes._ (cm^3^/g)
Na-X-LS	734	693.194	27.501	0.302	0.020
Na-X-TS	671	626.911	36.344	0.314	0.052
Na-A-LS	8	2.123	3.981	0.001	0.014
Na-A-TS	19	3.542	10.922	0.002	0.005

## Data Availability

The data presented in this study are available on request from the corresponding author.

## Supplementary Materials

The following are available online at https://www.mdpi.com/1996-1944/14/6/1413/s1, Figure S1. Mineral phase composition of commercial Zeolite 13X (Na-X), Figure S2. Mineral phase composition of commercial Linde A (Na-A) zeolite. Table S1. Chemical composition of commercial Zeolite 13X (Na-X) and Linde A (Na-A). Table S2. Textural parameters of commercial zeolites Na-X and Na-A.
