# Supplementary material for: Recycling of Waste Solution after Hydrothermal Conversion of Fly Ash on a Semi-Technical Scale for Zeolite Synthesis
Source: Materials (Basel). 2021 Mar 15;14(6):1413. doi: 10.3390/ma14061413 (PMC8000054; doi:10.3390/ma14061413)
Supplement: Supplementary file 1 [file materials-14-01413-s001.pdf]

materials

MDPI

Supplementary Materials

Recycling of Waste Solution after Hydrothermal Conversion of Fly Ash on a Semi-Technical Scale for Zeolite Synthesis

Rafał Panek [1,*], Jarosław Madej [1], Lidia Bandura [1], and Grzegorz Słowik [2]

[1] Department of Geotechnical Engineering, Civil Engineering and Architecture Faculty, Lublin University of Technology, Nadbystrzycka 40, 20-618 Lublin, Poland; r.panek@pollub.pl (R.P.); j.madej@pollub.pl (J.M.); l.bandura@pollub.pl (L.B.)
[2] Department of Chemical Technology, Institute of Chemical Sciences, Faculty of Chemistry, Maria Curie-Sklodowska University in Lublin, Maria Curie-Sklodowska Sq. 3, 20-031 Lublin, Poland; grzegorz.slowik@poczta.umcs.lublin.pl
* Correspondence: r.panek@pollub.pl

In order to compare the zeolites Na-X and Na-A synthesized on laboratory and technical scale ccommercial forms of Na-X and Na-A zeolites were purchased from Sigma-Aldrich (USA) and characterized in terms of chemical composition, mineral phases composition, and textural parameters using the procedures described in the section 2.3 in the Manuscript.

Table S1. Chemical composition of commercial Zeolite 13X (Na-X) and Linde A (Na-A)

	**Zeolite 13X (Na-X)**	**Linde A (Na-A)**
	**[%]**	
**Na_2O**	12.73	17.25
**MgO**	nd	nd
**Al_2O_3**	23.08	28.89
**SiO_2**	44.73	38.90
**K_2O**	0.03	0.08
**CaO**	0.02	0.03
**TiO_2**	nd	0.01
**Fe_2O_3**	0.02	0.02
**LOI**	19.24	16.82

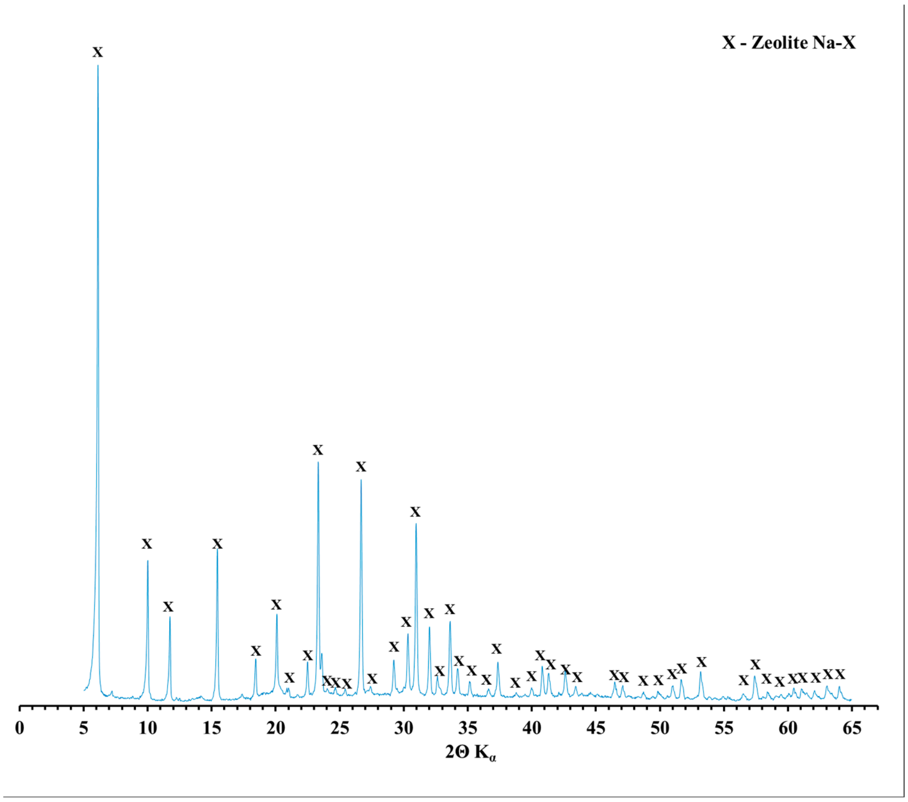

Figure S1. Mineral phase composition of commercial Zeolite 13X (Na-X)

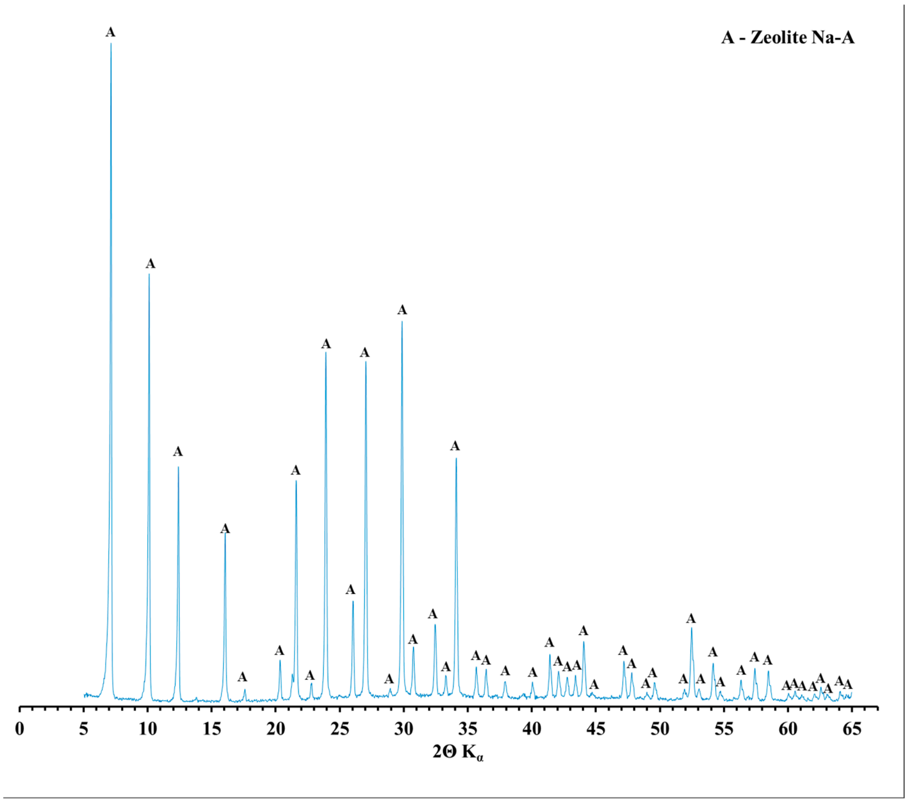

Figure S2. Mineral phase composition of commercial Linde A (Na-A) zeolite

Table S2. Textural parameters of commercial zeolites Na-X and Na-A

Zeolite	S_{BET} [m²/g]	$S_{mic.}$[m²/g]	$S_{mes.}$ [m²/g]	$V_{mic.}$ [cm³/g]	$V_{mes.}$ [cm³/g]
Zeolite 13X (Na-X)	646	615.938	2.142	0.238	0.006
Linde A (Na-A)	7	1.723	4.291	0.011	0.008
